# Renal Dysfunction Phenotypes in Patients Undergoing Obesity Surgery

**DOI:** 10.3390/biom13050790

**Published:** 2023-05-03

**Authors:** Pedro R. Pereira, João Pereira, Patrícia C. Braga, Sofia S. Pereira, Mário Nora, Marta Guimarães, Mariana P. Monteiro, Anabela Rodrigues

**Affiliations:** 1 Department of Nephrology, Centro Hospitalar de Trás-os-Montes e Alto Douro (CHTMAD), 5000-508 Vila Real, Portugal; 2 UMIB—Unit for Multidisciplinary Research in Biomedicine, ICBAS—School of Medicine and Biomedical Sciences, University of Porto, Rua Jorge Viterbo Ferreira 228, 4050-313 Porto, Portugal; 3 ITR—Laboratory of Integrative and Translocation Research in Population Health, Rua das Taipas 135, 4050-600 Porto, Portugal; 4 Department of General Surgery, Hospital São Sebastião, Centro Hospitalar de Entre o Douro e Vouga, Rua Dr. Cândido Pinho, 4050-220 Santa Maria da Feira, Portugal; 5 Department of Nephrology, Centro Hospitalar Universitário de Santo António, 4099-001 Porto, Portugal

**Keywords:** obesity, fatty kidney, obesity-related glomerulopathy, albuminuria, proteinuria

## Abstract

Obesity surgery candidates are at an increased risk of kidney injury, but pre-operative evaluation usually neglects kidney function assessment. This study aimed to identify renal dysfunction in candidates for bariatric surgery. To reduce the sources of bias, subjects with diabetes, prediabetes under metformin treatment, neoplastic or inflammatory diseases were excluded. Patients’ (n = 192) average body mass index was 41.7 ± 5.4 kg/m^2^. Among these, 51% (n = 94) had creatinine clearance over 140 mL/min, 22.4% (n = 43) had proteinuria over 150 mg/day and 14.6% (n = 28) albuminuria over 30 mg/day. A creatinine clearance higher than 140 mL/min was associated with higher levels of proteinuria and albuminuria. Univariate analysis identified sex, glycated hemoglobin, uric acid, HDL and VLDL cholesterol as being associated with albuminuria, but not with proteinuria. On multivariate analysis, glycated hemoglobin and creatinine clearance as continuous variables were significantly associated with albuminuria. In summary, in our patient population prediabetes, lipid abnormalities and hyperuricemia were associated with albuminuria, but not with proteinuria, suggesting different disease mechanisms might be implicated. Data suggest that in obesity-associated kidney disease, tubulointerstitial injury precedes glomerulopathy. A significant proportion of obesity surgery candidates present clinically relevant albuminuria and proteinuria along with renal hyperfiltration, suggesting that routine pre-operative assessment of these parameters should be considered.

## 1. Introduction

Obesity is a public health problem with significant implications for individuals and societies, and its worldwide prevalence is projected to continue to increase [1]. Obesity can lead to kidney injury through direct and indirect mechanisms and is an independent risk factor for the development and progression of chronic kidney disease (CKD) [2,3]. The prevalence of obesity-related glomerulopathy (ORG), which is characterized by proteinuria, glomerulomegaly, slowly progressive glomerulosclerosis and renal functional decline, rises with the prevalence of obesity [4]. 

The pathways through which obesity progresses to CKD are not well understood. Besides glomerular hyperfiltration, which has been hypothesized as the main mechanism linking metabolically unhealthy obesity to renal injury [5], studies have shown that in the setting of obesity there is also an excess tubular sodium reabsorption [6], an overactivation of the renin-angiotensin-aldosterone system [7] and an increased activation of the renal sympathetic nervous system, which are also likely to play a role in the disease pathology. Individuals with obesity have been found to have a significantly higher kidney weight [8], which has been attributed to compensatory hypertrophy of individual nephrons, but also to ectopic lipid accumulation in the kidney, the “fatty kidney” [9]. Lipid components have been shown to accumulate intra- and extracellularly in patients with obesity [8], contributing to structural and functional changes in mesangial cells, podocytes and proximal tubular cells [9], and are associated with ensuing obesity-related kidney disease. There is a gap in the knowledge concerning the pathways of glomerular versus tubular lesion in obesity. 

Bariatric surgery, an effective option for treatment-refractory obesity, requires extensive peri-operative assessment of patients [10]. Despite bariatric patients’ high risk of renal dysfunction given obesity-related glomerulopathy and other mechanisms, kidney function characterization in most surgical treatment of obesity programs is usually limited to pre-surgical serum urea and creatinine measurements. There are currently no practice guidelines regarding how to evaluate kidney function in patients pursuing bariatric surgery. In this study, we performed a detailed characterization of kidney function markers in patients with obesity attending a public academic center for surgical treatment of obesity. We aimed to study how common renal involvement is in bariatric surgery candidates and to evaluate clinical phenotypes with a focus on biomarkers of glomerular versus tubular lesion.

## 2. Materials and Methods

### 2.1. Study Population and Inclusion Criteria

The subjects enrolled in this study (n = 242) received care at a single non academic public center for the surgical treatment of obesity—Centro Hospitalar de Entre Douro e Vouga(CHEDV). The authors included adults who were eligible for surgical treatment of obesity with a body mass index (BMI) higher than 40 kg/m^2^ or with a BMI higher than 35 kg/m^2^ in the presence of obesity-related comorbidities. Patients were excluded from the study if they had diabetes mellitus (HbA1c > 6.5% or were under anti-diabetic drugs other than metformin regardless the HbA1c value (n = 25), prediabetes treated with metformin (5.7 > HbA1c > 6.5% and taking metformin, n = 14), neoplastic diseases (n = 0), or any inflammatory conditions (n = 0). Patients with a 24 h urine collection volume under 800 mL (n = 11), in order to avoid putative errors in 24 h urine collection that could affect creatinine clearance calculation, were also excluded, leading to the final number of patients included (n = 192). The protocol was submitted and granted approval by the Institutional Review Board (CA-014/20-Ot_MP/CC) before the study was started.

### 2.2. Data Acquisition

Data concerning age, gender, weight, BMI, blood pressure, hypertension status and hypertension therapy, diabetes status and diabetes therapy, dyslipidemia status, complete blood count, serum creatinine, serum urea, sodium, potassium, chloride, uric acid levels, fasting glucose, glycated hemoglobin, total serum proteins, lipid profile (total cholesterol, LDL cholesterol, HDL cholesterol, triglycerides, non-LDL cholesterol), urinalysis, and measurements of albuminuria, proteinuria and non-indexed creatinine clearance based on a 24 h urine collection, were acquired for each patient. The 2021 CKD-EPI Creatinine [11] was used to calculate the estimated glomerular filtration rate (GFR) in this patient population.

The patients were instructed to collect 24 h urine on two consecutive days before the blood sampling. The patients were given oral and written instructions on how to perform a 24 h urine collection. Urinary albumin, urinary protein and creatinine concentrations were measured on each collection. The 24 h creatinine clearance rate was calculated using the measured serum creatinine concentration and the urine creatinine concentration of the 24 h urine and was not indexed to body surface area (BSA). The urinary albumin-to-protein ratio (uAPR) was calculated by dividing the 24 h urine concentrations of albumin and protein for each patient.

Patients were categorized into clinically significant levels of albuminuria (higher or lower than 30 mg/24 h) and proteinuria (higher or lower than 150 mg/24 h) as defined by the KDIGO guidelines [12]. A threshold of creatinine clearance of 140 mL/min was used to classify patients as having significant hyperfiltration [13]. A threshold value of 0.15 (median value) was used to study patients according to uAPR.

### 2.3. Statistical Analysis

All data presented are expressed as mean ± standard deviation (SD), unless otherwise specified. The Shapiro–Wilk test was used to determine the normality of the groups. A comparison of independent groups was carried out by using either an unpaired *t*-test or a Mann–Whitney U test, depending on the normality. To compare 2 or more nominal variables, we used a χ^2^ test. A *p*-value < 0.05 was considered statistically significant. To assess relative risk increase / adjusted odds ratios a binary logistic regression model was employed using SPSS version 28.0, either by using single or combined variables. Statistical analysis was carried out using GraphPad (Prism; Version 8.0.1) and SPSS (IBM; Version 28.0) for Windows.

## 3. Results

### 3.1. Subjects

This patient cohort (n = 192) comprised 153 (79.7%) female patients and 39 (20.3%) male patients, with an average age of 42 ± 11 years and an average BMI of 41.7 ± 5.4 kg/m^2^. The clinical and biochemical characteristics of patients are depicted in Table 1. Dyslipidemia was present in 57.3% (n = 110) of patients and hypertension was present in 36.5% (n = 70). Average glycated hemoglobin was 5.4 ± 0.4% and prediabetes status was present in 36.5% (n = 40) patients. For patients with hypertension (n = 70, 36.5%), 38 (54.3%) were under therapy with angiotensin-converting enzyme (ACE) inhibitors or angiotensin II receptor blockers (ARBs). 

### 3.2. Markers of Kidney Injury

The average serum creatinine was 0.8 ± 0.1 mg/dL and the average creatinine clearance was 145 ± 44 mL/min, while the average GFR estimation through the 2021 CKD-EPI Creatinine was 104 ± 14 mL/min/1.73 m^2^, depicting a weak positive relation with the 24 h creatinine clearance (r = 0.3505, *p* < 0.001). Creatinine clearance was higher than 140 mL/min in 51% (n = 98) of patients and higher than 170 mL/min in 27.6% (n = 53) of patients. Most patients had a creatinine clearance above 90 mL/min (n = 178, 92.7%) (KDIGO G1). The creatinine clearance was below 90 mL/min in 7.3% (n = 14) and, from these, 11 patients had creatinine clearance between 60–90 mL/min (KDIGO G2) and 3 patients had a creatinine clearance between 45–60 mL/min (KDIGO G3a) [12]. Clinically significant proteinuria (above 150 mg/day) was present in 22.4% (n = 43) of patients; such patients had an average proteinuria of 220 ± 96 mg/day. Albuminuria over 30 mg/day occurred in 27 patients (14.1%) (KDIGO A2) and 1 patient (0.5%) had albuminuria higher than 300 mg/day (KDIGO A3) [12].

The uAPR averaged 0.2 ± 0.1, with most patients showing levels between 0.1 and 0.3 (n = 114, 59.4%). Table 2, Table 3, Table 4, Table 5 and Table 6 show clinical and laboratory variables according to the presence of markers of kidney damage.

### 3.3. Clinically Relevant Associations

A creatinine clearance higher than 140 mL/min was associated with higher levels of proteinuria and albuminuria (Table 2). 

Albuminuria was associated with higher levels of glycated hemoglobin (5.6 ± 0.3% versus 5.3 ± 0.4%; <0.001), while proteinuria values did not show a significant association with glycated hemoglobin (Table 3 and Table 4). 

Among the population with prediabetes (n = 40, 20.8%), the percentage of patients with albuminuria was 25% (n = 10), while among the population with euglycemia (n = 152), this percentage was of 11.8% (n = 18) (Table 4). 

Similarly to glycated hemoglobin, albuminuria was associated with significantly higher values of serum uric acid compared to patients with normal albuminuria levels (6.5 ± 1.3 mg/dL vs. 5.5 ± 1.3 mg/dL, *p* < 0.05, Table 4), while proteinuria values did not show a significant association with serum uric acid (Table 3). 

Several lipid parameters also showed significant associations with albuminuria, but not with proteinuria; HDL cholesterol was significantly lower in patients with albuminuria (45.1 ± 9.7 mg/dL vs. 49.9 ± 11.2 mg/dL, *p* < 0.05), while levels of LDL cholesterol (143.7 ± 37.5 mg/dL vs. 130.6 ± 35.4 mg/dL, *p* < 0.05), VLDL cholesterol (32.0 ± 15.2 mg/dL vs. 24.0 ± 11.8 mg/dL, *p* < 0.05), triglycerides (160.1 ± 76.1 mg/dL vs. 120.1 ± 59.1 mg/dL, *p* < 0.05) and non-LDL cholesterol (158.4 ± 35.9 mg/dL vs. 141.9 ± 34.9 mg/dL, *p* < 0.05) were significantly higher in patients with albuminuria. 

The average atherogenic index was significantly higher in patients with proteinuria, while other lipid parameters did not show significant differences between patients with or without clinically significant proteinuria. 

Clinical and biochemical characteristics according to uAPR higher or lower than 0.15 are presented (Table 5). Proportionally higher levels of proteinuria relative to albuminuria were not associated with glycated hemoglobin or with lipid parameters.

**Figure 1:**
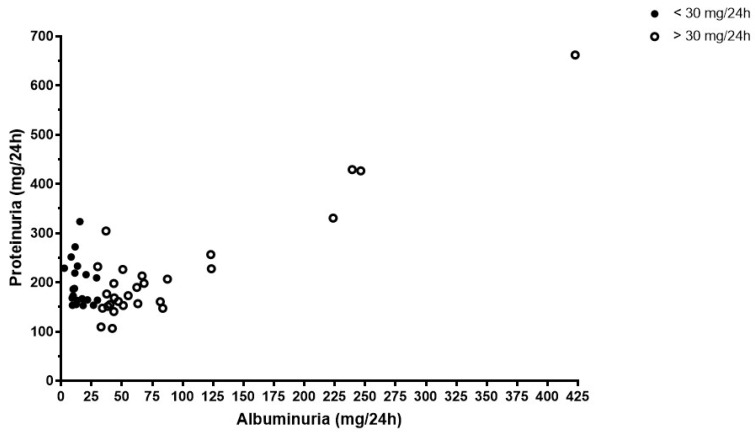
Levels of proteinuria and albuminuria in patients with albuminuria higher than 30 mg/day or proteinuria higher than 150 mg/day.

Figure 1 shows the distribution of patients according to levels of proteinuria and albuminuria in patients with clinically significant levels of any of these parameters.

Males had significantly worse metabolic and renal function parameters compared to females, showing higher levels of albuminuria, proteinuria, creatinine clearance, dyslipidemia, hypertension and prediabetes. This led us to analyze males and females as two distinct populations. Proteinuria higher than 150 mg/day occurred in 35.9% of males versus 19.0% of females (*p* < 0.05); albuminuria higher than 30 mg/day occurred in 35.6% of males compared to 9.2% of females (*p* < 0.001); creatinine clearance over 140 mL/min was observed in 79.5% of males versus 43.8% of females (*p* < 0.001). In male patients, but not in female patients, proteinuria higher than 150 mg/day was associated with significantly higher BMI, compared to male patients with proteinuria under 150 mg/day (46 ± 19 kg/m^2^ vs. 41 ± 5 kg/m^2^; *p* < 0.05). Male patients had higher uAPR compared to female patients, with levels above 0.15 in 66.7% of male patients compared to 44.4% of female patients (*p* < 0.05). 

Univariate analysis identified sex, glycated hemoglobin, uric acid, HDL cholesterol and VLDL cholesterol as being associated with albuminuria, but not with proteinuria. Creatinine clearance was associated with higher values of both albuminuria and proteinuria. On multivariate analysis, glycated hemoglobin and creatinine clearance as continuous variables were found to be significantly associated with albuminuria (Table 6). For each unitary increase in the level of glycated hemoglobin, there was a 5.283 times higher chance of albuminuria, while for each unitary increase in the level of creatinine clearance, there was a 1.010 times higher chance of albuminuria.

## 4. Discussion

Obesity has shown to play an important role in CKD development and progression independently of its etiology [9]. Qualification criteria for bariatric surgery include BMI and obesity-related comorbidities, but kidney dysfunction is most often overlooked. In this study we aimed to depict the prevalence of kidney injury markers in candidates to bariatric surgery without diabetes or other known chronic inflammatory diseases. 

Importantly, several methodological issues were considered. Measurements were based on a 24 h urine collection; creatinine clearance was not scaled for BSA to avoid the systematic underestimation of GFR that happens with BSA indexing, particularly noticeably in the population of patients with obesity [14,15]. A severe increase in creatinine clearance (more than 170 mL/min) was present in 27.6% of patients and it was higher than 140 mL/min in more than half of the population. These findings are consistent with previous studies showing that renal hyperfiltration is a characteristic feature in obesity-related renal disease [16,17] that appears in the early stages of higher than normal adiposity and even for BMIs under 30 kg/m^2^ [18,19]. Another important observation was the weak correlation between measured 24 h creatinine clearance and the estimated GFR using the 2021 CKD-EPI Creatinine (145 ± 44 mL/min vs. 104 ± 14 mL/min/1.73 m^2^) in our patient population, which supports findings from recent studies suggesting this equation might underestimate GFR in patients with obesity [20]. As expected, both proteinuria and albuminuria were higher in patients with higher creatinine clearances [21,22]. Several factors have been shown to contribute to increased albumin excretion in patients with obesity [4,23,24]. However, there is a gap in the knowledge related to renal tubular injury and to the qualitative composition of proteinuria. Beyond the commonplace of glomerulomegaly and hyperfiltration, obesity-related kidney disease may have tubule-centric mechanisms of injury, as it occurs in diabetes mellitus [25]. 

Indeed, in our study, the rate of patients with proteinuria higher than 150 mg/day was 22.4%, and the rate of patients with albuminuria higher than 30 mg/day was 14.6%, findings that are consistent with previous studies in populations with obesity, but with higher proportions [26,27,28,29] this supports that pre-operative assessment should include a panel of renal biomarkers. 

It should be highlighted that, to reduce the risk of bias, our study did not include patients with diabetes nor patients with prediabetes under metformin treatment. Nevertheless, albuminuria levels above 30 mg/day were associated with higher levels of glycated hemoglobin than in patients with albuminuria lower than 30 mg/day, and multivariate analysis showed glycated hemoglobin to significantly impact albuminuria levels. Prediabetes has been shown to be modestly associated with incident CKD [30] and studies have shown that it may have a significant role in the development of albuminuria and hyperfiltration [31,32,33]. Other studies support that some of the pathologic changes that are characteristic of diabetic kidney disease may be already present in the prediabetic state [34,35]. In our patient cohort, prediabetes was present in 40 (20.8%) patients and, from these, 25% had albuminuria, while among the euglycemic population (n = 152), this figure was of 11.8%. It has been suggested that there might be a synergistic effect between obesity-associated renal changes and prediabetes-associated renal changes, lowering the thresholds for the occurrence of kidney injury that is common to both pathologies in this patient population [36,37]. 

Several lipid abnormalities were shown to correlate with clinically relevant albuminuria. Lower levels of HDL cholesterol and higher levels of LDL cholesterol, VLDL cholesterol, triglycerides, non-HDL cholesterol and higher atherogenic index were associated with albuminuria higher than 30 mg/dL. In contrast to albuminuria, apart from the atherogenic index, these differences did not correlate with significant proteinuria or significant creatinine clearance. Dyslipidemia has also been associated with GFR decline [38] and increased incidence of albuminuria [39] in several previous studies. Alterations in lipid metabolism are being increasingly recognized as important factors in ectopic lipid accumulation in the kidney, known as “fatty kidney”, as well as increased oxidative stress, inflammation, and fibrosis [4]. In ORG, altered lipid metabolism has been shown to promote the deposition of triglycerides and cholesterol esters in the kidney, leading to maladaptive changes in renal cells [40,41]. Ectopic lipid accumulation compromises glomerular integrity and has been associated with albuminuria [42] and fibrosis [4], with changes in mesangial cells [9] and in podocytes [43].

Elevated serum uric acid levels have been shown to predict the development of albuminuria in type 1 diabetes [44], but the causal relationship between uric acid levels and CKD or the benefits of treating hyperuricemia in CKD are still a matter of debate [45,46]. In our study, uric acid levels were also significantly higher in patients with albuminuria over 30 mg/day. As in the previously described lipid abnormalities, this difference was noticed to be associated with increased albuminuria, but not with increased proteinuria. Higher uAPR was also associated with significantly higher uric acid levels.

The average uAPR in our patient population was 0.18 ± 0.12, with most patients showing levels between 0.1 and 0.3 (n = 114, 59.4%). To the best of our knowledge, there are no previous reports on uAPR in patients with obesity. As abovementioned, several variables were shown to have a distinctive impact on the levels of proteinuria and albuminuria. Several studies have proposed that the origin of urinary proteins can be inferred by measuring the uAPR and, in kidney diseases with significant proteinuria, a uAPR of <0.4 can predict primary tubulointerstitial, with a sensitivity and specificity of 88% and 99%, respectively [47,48,49]. In patients with obesity, these qualitative differences in protein excretion between patients (Figure 1) may reflect different types of early kidney involvement which may be related to the patient’s distinct metabolic signatures in the setting of obesity. We hypothesize that patients with significant lipid abnormalities, hyperuricemia and higher levels of glycated hemoglobin, might have a more glomerular-centered kidney injury in early disease and, therefore, present with higher rates of albuminuria, while, patients without these metabolic characteristics may develop primarily tubulointerstitial changes in early course of disease, with excretion of higher percentages of other urinary proteins, and only in later stages progress with glomerular dysfunction. While albuminuria is a marker of glomerular injury, non-albuminuric proteinuria may reflect tubular excretion and tubulointerstitial inflammation/injury [50,51]. Since albuminuria and proteinuria are interconnected, the biomarker urinary albumin-to-protein ratio (uAPR) is an opportunity to evaluate the proportional strength of such injury pathways. The tracks of tubule-glomerular and tubule-interstitial crosstalk in the pathophysiology of ”fatty kidney”, as well as the contribution of local fat to renal disease progression, deserve further investigation.

Several significant differences were noticed in renal function parameters between male and female participants. Male patients had significantly higher rates of proteinuria, albuminuria, uAPR and creatinine clearance compared to female patients. Obesity-related renal function differences between males and females have been reported in previous studies, showing conflicting results [52,53]. In our study these differences may reflect the uneven gender distribution of patients undergoing bariatric surgery, with men with obesity being less prone to seek earlier medical aid or consider surgical treatment [54]. 

This study allowed us to demonstrate that a significant proportion of bariatric surgery candidates present renal hyperfiltration along with clinically relevant albuminuria and proteinuria. Moreover, the data suggest that in obesity-associated kidney disease, tubulointerstitial injury precedes glomerulopathy, which is an important addition to preexisting knowledge. The significant prevalence of kidney injury we observed in our study shows kidney function should be evaluated in bariatric surgery candidates, something which is not routinely performed in most bariatric surgery programs. Patients with abnormal kidney function may deserve a long-term follow-up targeting renal function, as is currently advised for patients undergoing bariatric surgery with other comorbidities including diabetes, obstructive sleep apnea syndrome, or hypertension.

The main limitation of our study is the fact that it was a cross-sectional study. Follow-up of these patients could provide valuable information on how renal parameters, namely creatinine clearance and hyperfiltration, evolve according to the obesity/metabolic status and specifically after bariatric surgery. Even though our sample size was limited, it is similar to other studies in this area and was sufficiently powered to depict robust statistical differences. As important strengths, our study evaluated renal function parameters through a 24 h urine collection, which increased our ability to analyze small variations in protein excretion, as opposed to the alternative use of a spot albumin-creatinine ratio, which is a good screening test for microalbuminuria, but performs poorly in determining quantitative albumin excretion rate or total proteinuria. Additionally, we used a non-indexed creatinine clearance calculation, and collected an extensive panel of biochemical variables, which allowed us to conduct a detailed characterization and broader analysis of our patient population. 

In conclusion, a significant proportion of patients with obesity have clinically relevant levels of albuminuria, proteinuria and hyperfiltration, as early markers of renal lesion. In patients with obesity, the concomitant presence of prediabetes, lipid abnormalities and hyperuricemia were associated with clinically relevant levels of albuminuria, but not with proteinuria, which could translate into different types of kidney compartment injury. Our results highlight that traces of tubulointerstitial injury also occur in obesity-related kidney disease, together with or even in advance of glomerular damage, which warrants further investigation.

## Figures and Tables

**Table 1:** Clinical and biochemical characteristics of the patient population.

		Total (n = 192)
Age (years)		42 ± 11
Sex, n (%) of females		153 (79.7%)
Weight (kg)		112 ± 18
BMI (kg/m^2^)		41.7 ± 5.4
Glycaemic Status	No diabetes, n (%)	152 (79.2%)
	Prediabetes (no metformin), n (%)	40 (20.8%)
HTN, n (%)		70 (36.5%)
Dyslipidaemia, n (%)		110 (57.3%)
Glucose (mg/dL)		92 ± 11
Glycated haemoglobin (%)		5.4 ± 0.4
Serum urea (mg/dL)		30.7 ± 7.8
Serum creatinine (mg/dL)		0.8 ± 0.1
Uric acid (mg/dL)		5.6 ± 1.3
Total cholesterol (mg/dL)		193.5 ± 36.0
HDL cholesterol (mg/dL)		49.2 ± 11.1
LDL cholesterol (mg/dL)		132.5 ± 35.9
VLDL cholesterol (mg/dL)		25.2 ± 12.6
Triglycerides (mg/dL)		126.0 ± 63,2
Non-LDL Cholesterol (mg/dL)		144.3 ± 35.4
Atherogenic index		0.4 ± 0.2
Total Proteins (mg/dL)		7.0 ± 0.3
Diuresis (mL)		1547.3 ± 569.6
Albuminuria (mg/day)		24.1 ± 43.6
Proteinuria (mg/day)		117.6 ± 76.1
Creatinine clearance (mL/min)		145.2 ± 44.1
uAPR		0.2 ± 0.1
Creatinine Clearance	<90 mL/min, n (%)	14 (7.3%)
	90–130 mL/min, n (%)	66 (34.4%)
	130–170 mL/min, n (%)	59 (30.7%)
	>170 mL/min, n (%)	53 (27.6%)
Proteinuria	<150 mg/24 h, n (%)	149 (77.6%)
	>150 mg/24 h, n (%)	43 (22.4%)
Albuminuria	<30 mg/24 h, n (%)	164 (85.4%)
	>30 mg/24 h, n (%)	28 (14.6%)
uAPR	<0.1, n (%)	54 (28.1%)
	0.1–0.3, n (%)	114 (59.4%)
	>0.3, n (%)	24 (12.5%)
	>0.15%	94 (49.0%)

Continuous variables are presented in mean ± standard deviation. Abbreviations: BMI, body mass index; HTN, hypertension; HDL, high density lipoprotein; VLDL, very-low density lipoprotein; uAPR, urinary albumin-to-protein ratio.

**Table 2:** Clinical and biochemical characteristics according to creatinine clearance higher or lower than 140 mL/min.

	Creatinine Clearance		*p*
	ClCr < 140 mL/min (n = 94)	ClCr > 140 mL/min (n = 98)	
Sex, n (%) of females	86 (91.5%)	67 (68.4%)	<0.001
HTA, n (%)	56 (59.6%)	66 (67.3%)	0.295
Dyslipidaemia, n (%)	46 (48.9%)	36 (36.7%)	0.109
Age, years	42.6 ± 11.9	41.6 ± 10.6	0.513
Weight, kg	106.6 ± 14.5	116.5 ± 19.9	<0.001
BMI, kg/m^2^	41.0 ± 4.5	42.0 ± 7.1	0.121
Glucose, mg/dL	90.6 ± 11.0	93.7 ± 11.7	0.046
HbA1c (%)	5.3 ± 0.4	5.4 ± 0.4	0.115
Urea, mg/dL	31.2 ± 8.0	30.26 ± 7.7	0.376
Creatinine, mg/dL	0.8 ± 0.2	0.8 ± 0.1	0.280
Uric acid, mg/dL	5.4 ± 1.2	5.78 ± 1.39	0.083
Total cholesterol, mg/dL	191.0 ± 33.7	195.9 ± 38.0	0.352
HDL cholesterol, mg/dL	50.7 ± 10.9	47.8 ± 11.1	0.074
LDL cholesterol, mg/dL	127.0 ± 33.0	137.8 ± 37.9	0.083
VLD cholesterol, mg/dL	25.5 ± 12.8	24.9 ± 12.5	0.797
Triglycerides, mg/dL	127.4 ± 64.2	124.6 ± 62.6	0.785
Non-LDL Cholesterol, mg/dL	140.4 ± 32.7	148.1 ± 37.6	0.221
Atherogenic index	0.4 ± 0.2	0.4 ± 0.3	0.577
Total proteins, mg/dL	7.0 ± 0.3	7.1 ± 0.3	0.028
Albuminuria, mg/day	18.3 ± 34.2	29.6 ± 50.7	<0.001
Proteinuria, mg/day	97.8 ± 64.9	136.6 ± 81.3	<0.001
uAPR	0.2 ± 0.1	0.2 ± 0.1	0.242

Continuous variables are presented in mean ± standard deviation. Abbreviations: BMI, body mass index; HbA1c, hemoglobin A1C; HTN, hypertension; HDL, high density lipoprotein; VLDL, very-low density lipoprotein; uAPR, urinary albumin-to-protein ratio; ns, non-significative.

**Table 3:** Clinical and biochemical characteristics according to proteinuria higher or lower than 150 mg/24 h.

	Proteinuria Category		*p*
	<150 mg/24 h (n = 149)	>150 mg/24 h (n = 43)	
Sex, n (%) of females	123 (83.2%)	29 (67.4%)	0.023
HTA, n (%)	98 (65.8%)	24 (55.8%)	0.281
Dyslipidaemia, n (%)	67 (45.0%)	15 (34.9%)	0.294
Age, years	41.9 ± 11.4	42.6 ± 11.0	0.725
Weight, kg	110.8 ± 15.9	114.5 ± 24.4	0.765
BMI, kg/m^2^	41.3 ± 5.8	42.2 ± 6.8	0.898
Glucose, mg/dL	91.6 ± 11.4	94.0 ± 11.4	0.214
HbA1c (%)	5.3 ± 0.4	5.4 ± 0.4	0.098
Urea, mg/dL	30.4 ± 7.6	32.0 ± 8.5	0.556
Creatinine, mg/dL	0.8 ± 0.1	0.8 ± 0.2	0.663
Uric acid, mg/dL	5.5 ± 1.3	5.9 ± 1.3	0.116
Total cholesterol, mg/dL	192.8 ± 36.2	196.1 ± 35.5	0.590
HDL cholesterol, mg/dL	50.0 ± 11.4	46.6 ± 9.8	0.066
LDL cholesterol, mg/dL	131.6 ± 36.0	135.4 ± 35.9	0.553
VLD cholesterol, mg/dL	24.5 ± 12.5	27.5 ± 12.9	0.128
Triglycerides, mg/dL	122.7 ± 62.7	137.3 ± 64.6	0.127
Non-LDL Cholesterol, mg/dL	142.8 ± 35.3	149.6 ± 35.5	0.229
Atherogenic index	0.4 ± 0.2	0.4 ± 0.3	0.04
Total proteins, mg/dL	7.0 ± 0.3	7.2 ± 0.4	0.04
Albuminuria, mg/day	13.7 ± 9.2	60.0 ± 81.6	<0.001
Creatinine clearance, mL/min	139.8 ± 39.1	163.9 ± 54.6	0.002
uAPR	0.2 ± 0.8	0.2 ± 0.2	0.220

Continuous variables are presented in mean ± standard deviation. Abbreviations: BMI, body mass index; HbA1c, hemoglobin A1C; HTN, hypertension; HDL, high density lipoprotein; VLDL, very-low density lipoprotein; uAPR, urinary albumin-to-protein ratio; ns, non-significative.

**Table 4:** Clinical and biochemical characteristics according to albuminuria higher or lower than 30 mg/24 h.

	Albuminuria Category		***p***
	<30 mg/24 h (n = 164)	>30 mg/24 h (n = 28)	
Sex, n (%) of females	139 (84.8%)	14 (50.0%)	<0.001
HTA, n (%)	108 (65.9%)	14 (50.0%)	0.137
Dyslipidaemia, n (%)	73 (44.5%)	9 (32.1%)	0.302
Age, years	42.0 ± 11.3	42.7 ± 11.1	0.753
Weight, kg	110.2 ± 15.6	120.4 ± 27.7	0.092
BMI, kg/m^2^	41.3 ± 5.6	43.1 ± 7.9	0.578
Glucose, mg/dL	92.0 ± 11.3	93.0 ± 12.1	0.553
HbA1c (%)	5.3 ± 0.4	5.6 ± 0.3	<0.001
Urea, mg/dL	30.7 ± 8.1	30.8 ± 5.8	0.887
Creatinine, mg/dL	0.8 ± 0.1	0.8 ± 0.1	0.202
Uric acid, mg/dL	5.5 ± 1.3	6.5 ± 1.3	<0.001
Total cholesterol, mg/dL	191.8 ± 35.5	203.5 ± 37.8	0.113
HDL cholesterol, mg/dL	49.9 ± 11.2	45.1 ± 9.7	0.046
LDL cholesterol, mg/dL	130.6 ± 35.4	143.7 ± 37.5	0.083
VLD cholesterol, mg/dL	24.0 ± 11.8	32.0 ± 15.2	0.003
Triglycerides, mg/dL	120.1 ± 59.1	160.1 ± 76.1	0.003
Non-LDL Cholesterol, mg/dL	141.9 ± 34.9	158.4 ± 35.9	0.023
Atherogenic index	0.3 ± 0.2	0.5 ± 0.2	<0.001
Total proteins, mg/dL	7.0 ± 0.3	7.2 ± 0.4	0.001
Proteinuria, mg/day	99.8 ± 47.8	221.6 ± 118.3	<0.001
Creatinine clearance, mL/min	141.0 ± 39.2	170.2 ± 60.7	0.001
uAPR	14.5 ± 7.4	36.5 ± 15.1	<0.001

Continuous variables are presented in mean ± standard deviation. Abbreviations: BMI, body mass index; HbA1c, hemoglobin A1C; HTN, hypertension; HDL, high density lipoprotein; VLDL, very-low density lipoprotein; uAPR, urinary albumin-to-protein ratio; ns, non-significative.

**Table 5:** Clinical and biochemical characteristics according to uAPR higher or lower than 0.15.

	uAPR		*p*
	uAPR < 0.15 (n = 98)	aAPR > 0.15 (n = 94)	
Sex, n (%) of females	85 (86.7%)	68 (72.3%)	0.013
HTA, n (%)	65 (66.3%)	57 (60.6%)	0.455
Dyslipidaemia, n (%)	45 (45.9%)	37 (39.4%)	0.384
Age, years	41.2 ± 10.6	43.0 ± 11.9	0.299
Weight, kg	109.4 ± 15.5	114.0 ± 20.4	0.100
BMI, kg/m^2^	41.0 ± 4.4	42.0 ± 7.3	0.247
Glucose, mg/dL	92.9 ± 12.1	91.4 ± 10.7	0.713
HbA1c (%)	5.3 ± 0.4	5.4 ± 0.4	0.338
Urea, mg/dL	30.1 ± 7.7	31.4 ± 7.9	0.345
Creatinine, mg/dL	0.8 ± 0.2	0.8 ± 0.1	0.693
Uric acid, mg/dL	5.4 ± 1.3	5.9 ± 1.4	0.014
Total cholesterol, mg/dL	190.0 ± 31.7	197.2 ± 39.8	0.170
HDL cholesterol, mg/dL	49.8 ± 11.6	48.6 ± 10.6	0.489
LDL cholesterol, mg/dL	128.0 ± 31.2	137.2 ± 39.8	0.075
VLD cholesterol, mg/dL	24.2 ± 11.7	26.3 ± 13.5	0.320
Triglycerides, mg/dL	120.8 ± 58.6	131.4 ± 67.5	0.315
Non-LDL Cholesterol, mg/dL	140.2 ± 30.4	148.6 ± 39.6	0.099
Atherogenic index	0.4 ± 0.3	0.4 ± 0.2	0.227
Total proteins, mg/dL	7.0 ± 0.3	7.1 ± 0.4	0.136
Albuminuria, mg/day	10.6 ± 5.9	38.1 ± 59.0	<0.001
Proteinuria, mg/day	116.8 ± 57.2	118.5 ± 92.1	0.174
Creatinine clearance, mL/min	139.9 ± 38.9	150.8 ± 48.5	0.145

Continuous variables are presented in mean ± standard deviation. Abbreviations: BMI, body mass index; HbA1c, hemoglobin; HTN, hypertension; HDL, high density lipoprotein; VLDL, very-low density lipoprotein; uAPR, urinary albumin-to-protein ratio; ns, non-significative.

**Table 6:** Univariate analysis for albuminuria and proteinuria according to several variables, and multivariate analysis for albuminuria according to significant variables.

	Albuminuria Univariate			Albuminuria Multivariate			Proteinuria Univariate		
Variable	HR (95% CI)		*p*	HR (95% CI)		*p*	HR (95% CI)		*p*
Age	1.006	(0.971–1.042)	0.752	-	-	-	1.005	(0.976–1.036)	0.723
Sex	5.56	(2.366–13.066)	<0.001	1.582	(0.426– 5.879)	0.494	2.394	(1.110–5.167)	0.026
BMI	1.051	(0.983–1.124)	0.148	-	-	-	1.023	(0.963–1.087)	0.457
Glucose	1.008	(0.974–1.043)	0.663	-	-	-	1.017	(0.989–1.047)	0.24
HbA1c	6.551	(2.181–19.678)	<0.001	5.283	(1.610–17.332)	0.006	2.229	(0.901–5.514)	0.083
Urea	1.001	(0.951–1.054)	0.967	-	-	-	1.025	(0.983–1.070)	0.245
Creatinine	2.045	(0.124–33.592)	0.616	-	-	-	2.92	(0.262–32.354)	0.384
CrCl	1.014	(1.004–1.023)	0.004	1.011	(1.001–1.022)	0.040	1.012	(1.004–1.021)	0.003
Uric Acid	1.72	(1.267–2.336)	<0.001	1.360	(0.877–2.107)	0.169	1.193	(0.928–1.533)	0.169
Total Cholesterol	1.009	(0.998–1.020)	0.115	-	-	-	1.003	(0.993–1.012)	0.588
HDL	0.957	(0.918–0.997)	0.037	0.999	(0.954–1.047)	0.979	0.97	(0.939–1.003)	0.077
LDL	1.01	(0.999–1.021)	0.076	-	-	-	1.003	(0.994–1.012)	0.542
Age	1.006	(0.971–1.042)	0.752	-	-	-	1.005	(0.976–1.036)	0.723
Sex	5.56	(2.366–13.066)	<0.001	1.582	(0.426– 5.879)	0.494	2.394	(1.110–5.167)	0.026
BMI	1.051	(0.983–1.124)	0.148	-	-	-	1.023	(0.963–1.087)	0.457
Glucose	1.008	(0.974–1.043)	0.663	-	-	-	1.017	(0.989–1.047)	0.24
HbA1c	6.551	(2.181–19.678)	<0.001	5.283	(1.610–17.332)	0.006	2.229	(0.901–5.514)	0.083
Urea	1.001	(0.951–1.054)	0.967	-	-	-	1.025	(0.983–1.070)	0.245
Creatinine	2.045	(0.124–33.592)	0.616	-	-	-	2.92	(0.262–32.354)	0.384
CrCl	1.014	(1.004–1.023)	0.004	1.011	(1.001–1.022)	0.040	1.012	(1.004–1.021)	0.003
Uric Acid	1.72	(1.267–2.336)	<0.001	1.360	(0.877–2.107)	0.169	1.193	(0.928–1.533)	0.169
Total Cholesterol	1.009	(0.998–1.020)	0.115	-	-	-	1.003	(0.993–1.012)	0.588
HDL	0.957	(0.918–0.997)	0.037	0.999	(0.954–1.047)	0.979	0.97	(0.939–1.003)	0.077
LDL	1.01	(0.999–1.021)	0.076	-	-	-	1.003	(0.994–1.012)	0.542

Abbreviations: BMI, body mass index; ClCr, Creatinine Clearance HbA1c, glycated hemoglobin; HTN, hypertension; HDL, high density lipoprotein; HR, hazard ration; LDL, low density lipoprotein; n.s, non-significative.

## Data Availability

The data presented in this study are available upon request from the corresponding author due to ethical concerns.
